# RADIOFREQUENCY THERAPY IN PATIENTS WITH OSTEOARTHRITIS AWAITING KNEE ARTHROPLASTY

**DOI:** 10.1590/1413-785220263404e300122

**Published:** 2026-07-24

**Authors:** Glaucus Cajaty dos Santos Martins, Ingrid Carvalho Andrade, Alexandre Peixoto de Mello, Bruno Parilha Coutinho, Vinicius Schott Gameiro

**Affiliations:** 1 Hospital Federal de Ipanema, Servico de Ortopedia e Traumatologia, Rio de Janeiro, RJ, Brazil.; 2 Universidade Federal do Estado do Rio de Janeiro (Unirio), Faculdade de Medicina, Rio de Janeiro, RJ, Brazil.; 3 Universidade Federal Fluminense (UFF), Faculdade de Medicina, Niterói, RJ, Brazil.

**Keywords:** Corticoid, Knee, Osteoarthritis, Radiofrequency, Corticoide, Joelho, Osteoartrite, Radiofrequência

## Abstract

**Objective:**

To evaluate the palliative use of conventional radiofrequency (RF) in patients awaiting total knee arthroplasty (TKA).

**Methods:**

Seventy-one knees with osteoarthritis and indication for TKA were evaluated and divided into two groups: Group 1 with 51 knees treated with RF and Group 2 with 20 patients treated with intra-articular corticosteroids. The primary outcome was pain response using the visual analog scale (VAS) at six months postoperatively. The secondary outcome was functional assessment using the WOMAC and KSS scales. The MCID (minimal clinically important difference) criterion was used. The influence of clinical and radiographic parameters on the procedural outcome was evaluated.

**Results:**

The RF group showed improvement in VAS at 6 months compared to the control group (χ2 test, p=0.0124). The RF group obtained a superior result than the control group using the WOMAC scale (χ2 test, p=0.044). The KSS, divided into knee and function subgroups, performed better than the control group (p=0.02 and p=0.048). Knee angulation and degree of osteoarthritis were associated with procedural prognosis (χ2 test, p=0.028, p=0.019).

**Conclusion:**

The RF group showed superior pain and functional improvements compared to the control group. Osteoarthritis severity and knee angulation influenced functional and pain outcomes. **Level of Evidence II; Comparative Prospective Study.**

## RESUMO

**Objetivo:**

Avaliar o uso paliativo de Radiofrequência (RF) convencional em pacientes que aguardam o procedimento de artroplastia total de joelho (ATJ).

**Métodos:**

Avaliados 71 joelhos com osteoartrite com indicação de ATJ divididos em 2 grupos: Grupo 1 com 51 joelhos tratados com RF e Grupo 2 com 20 pacientes tratados com corticoide intra-articular. O desfecho primário foi a resposta da dor pela escala visual analógica (EVA) ao sexto mês pós-operatório. O desfecho secundário foi a avaliação funcional pelas escalas WOMAC e KSS. Utilizado o critério de MCID (minimal clinically important difference). Avaliou-se a influência de parâmetros clínicos e radiográficos no resultado do procedimento.

**Resultados:**

O grupo RF comparado ao controle teve melhora do EVA ao final de 6 meses, teste χ2 (p=0,0124). O grupo RF obteve resultado superior ao controle pela escala WOMAC (teste χ2, p=0,044). O KSS dividido em subgrupos joelho e função obteve desempenho superior ao controle (p=0,02 e p=0,048). Angulação do joelho e grau de osteoartrite mostraram relação com prognóstico do procedimento (teste χ2, p=0,028, p=0,019).

**Conclusão:**

O grupo RF apresentou melhora da dor e funcional superiores ao grupo controle. Gravidade da artrose e angulação do joelho influenciaram os resultados funcionais e da dor. **Nível de Evidência II; Estudo Prospectivo Comparativo.**

## INTRODUCTION

The demand for knee arthroplasty procedures (TKA) in the public health system has progressively increased over the last few decades^1-4^. Various therapeutic modalities, including the use of radiofrequency therapy (RF) in the knee^3-10^, have been utilized palliatively while awaiting TKA.

Radiofrequency therapy in the knee involves the use of thermal energy generated by an electrode acting at the level of the genicular nerves. This leads to functional deactivation, preventing the transmission of painful stimuli from joint areas of the knee from reaching the cerebral sensory cortex, thereby blocking the sensation of pain ^6-9^.

In meta-analysis studies^11-14^, RF has proven effective in pain control for knee osteoarthritis in patients with poor clinical conditions to undergo TKA^1-9^. In public health, the waiting time for TKA can reach up to three years. This period inflicts considerable suffering on the patient. Palliative pain measures need to be adopted until the definitive surgery^4,5^.

In the reviewed literature, no studies were identified that exclusively address the use of RF in individuals indicated for TKA but who do not undergo it due to unavailability of the prosthesis.

A comparison was made between RF and the classical treatment of intra-articular corticosteroid injection^10^ in individuals with OA indicated for TKA. Parameters of the Visual Analog Scale for Pain (VAS) and functional WOMAC and KSS were evaluated^15,16^.

It should be emphasized the original aspect of this work in our environment and the potential contribution of the research results to the adoption of this palliative therapy in patients awaiting TKA.

## METHODOLOGY

This research adhered to the guidelines of the Helsinki Declaration and was approved by the research ethics committee of our hospital (CEP 5.071.669). This article constitutes a comparative and prospective cohort study.

A total of 61 individuals (55 women and 6 men) were selected from the knee outpatient clinic of a public institution with an indication for TKA. There was an expectation to wait at least one year on the waiting list.

The RF group was formed by 51 knees, and another 20 participants constituted the control group with intra-articular corticosteroid treatment. The research was conducted from January 2022 to December 2023. The authors initially sought to select the study and control groups in a 2:1 ratio.

Inclusion criteria: individuals over 50 years of age, with radiographic primary osteoarthritis, a visual analog scale (VAS) score of 5 or higher, no improvement with conservative treatment, and no previous open surgeries on the knee.

Exclusion criteria: rheumatological diseases, radiculopathies, use of anticoagulants, previous intra-articular injections with corticosteroids or hyaluronic acid within a period of less than six months.

The sample was based on works by El-Hakeim et al^3^ and Konya et al^17^ which determined that 30 patients would be sufficient to detect changes in the VAS (primary outcome) in 80% of cases with a significance level of 5%.

Pain was measured using the visual analog scale (VAS) from 0 to 10. Success was considered as a result of a reduction in pain equal to or greater than 50% after treatment.

Functional impairment was measured using the Western Ontario and McMaster Universities Osteoarthritis Index (WOMAC)^15^ which evaluates three domains: pain; stiffness; function. The minimal clinically important difference (MCID) criterion was employed (minimal clinically important difference)^15^, which indicates the minimum value representing a substantial improvement perceived by the patient. In the case of WOMAC and its subgroups, this would represent a variation of 16% in relation to the value obtained prior to the procedure. In the case of a successful outcome, the functional improvement value of the MCID must be achieved or exceeded.

The Knee Society Score (KSS)^16^ was also employed. This scale combines subjective and objective information and separates the knee score (which evaluates pain, stability, and range of motion) from the functional score (ability to walk, ascend, and descend stairs). The MCID was the minimum improvement of 9 points in the knee criterion and 10 points in the function criterion^16^.

Pain according to the VAS scale was assessed at the pre-intervention moment, the first month, and the sixth month after the procedure. A comparison was made between the control and RF groups. The WOMAC and KSS scales were evaluated similarly to the VAS.

Data were collected regarding age, gender, body mass index (BMI), duration of the disease, radiographic assessment of osteoarthritis according to the Kelgren and Alhback classifications, tibiofemoral radiographic alignment, and pre-treatment pain intensity. The same parameters were analyzed in the RF group regarding their prognostic value concerning the results. To allow for categorical statistical evaluation, types I to III of the Kelgren classification were grouped as mild or moderate involvement, and type IV as severe. Based on a previous study^18^, types I to III of Ahlback and types IV and V were divided into two different groups, which would present distinct morphopathological changes. A tibiofemoral alignment of 10 degrees or more was considered pathological valgus, neutral between 1 and 9 degrees, and varus at or below zero degrees. Due to there being only three cases in the neutral category, these were discarded for better statistical significance. The analysis and collection of results were performed by a physician not involved in the procedures.

**Figure 1:**
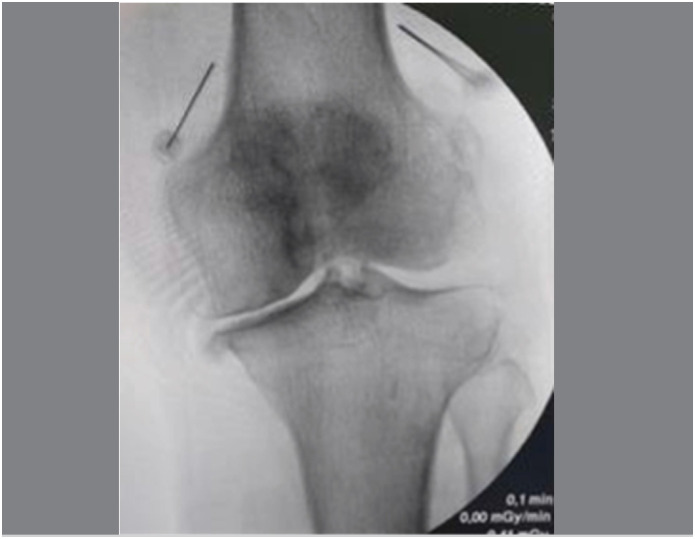
Positioning in AP radiography for thermoregulation of the superior medial genicular nerve and superior lateral genicular nerve. AP x-ray incidence of medial and lateral superior genicular nerves radiofrequency.

Source: Author's collection (2024).

**Figure 2:**
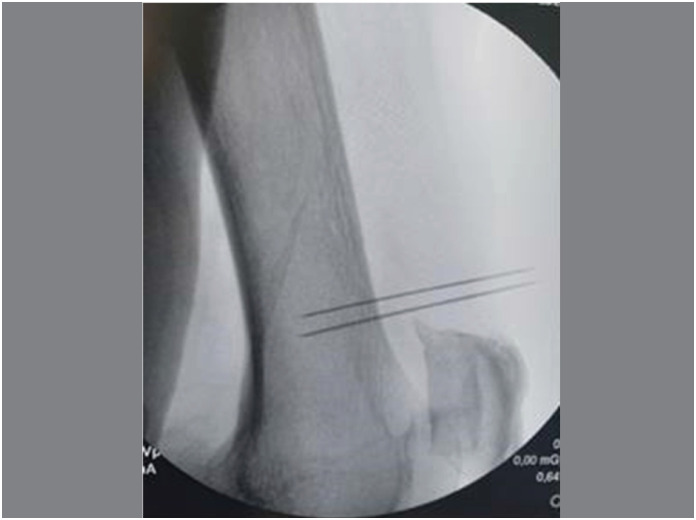
Positioning in Profile radiography for thermoregulation of the superior medial genicular nerve and superior lateral genicular nerve. rofile x-ray incidence of medial and lateral superior genicular nerves radiofrequency.

Source: Author's collection (2024).

**Figure 3:**
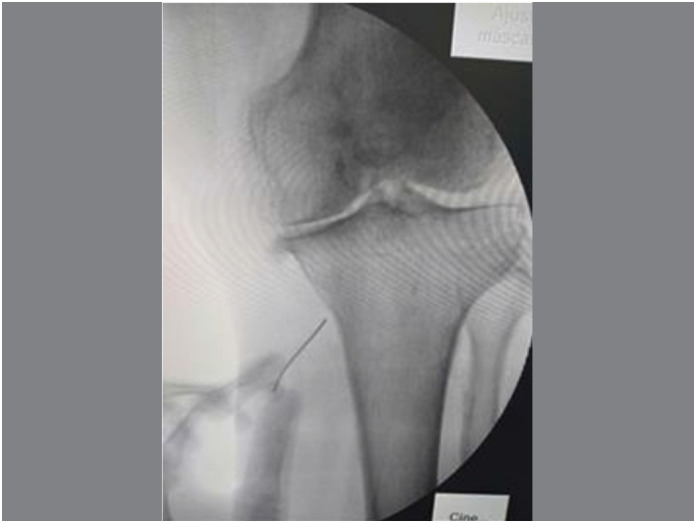
Positioning in AP incidence for thermoregulation of the inferior medial genicular nerve. AP x-ray incidence of medial inferior genicular nerve radiofrequency.

Source: Author's collection (2024).

- Corticosteroid Injection Technique: following classical methodology in the literature with the use of depot corticosteroid^10^.
- RF Technique: With methodology already described in the literature^1-5^, conventional cannulas were used. The patient was in a supine position, asepsis and antisepsis were performed, operative fields were placed, the knee was flexed at 15 degrees, under image intensifier control, infiltration of lidocaine was performed in the skin, subcutaneous tissue, muscle, and periosteum of the medial and lateral metaphyseal-diaphyseal junction of the distal femur and medial proximal tibia (Figures 1, 2, and 3). The inferior lateral genicular nerve is not approached due to the risk of compromising the fibular nerve. RF cannulas of 10 cm x 22 Gauge were used. Sensory stimulation test at 50Hz with a threshold below 0.6V. To avoid motor impairment, stimulation was performed at 2Hz and 2.0V, and muscle contractions should not occur. Through the cannulas, 2ml of lidocaine was infiltrated, and the cannula was heated to 70 degrees Celsius for 150 seconds.

### Statistical Analysis

The comparison of baseline variables between the groups (RF and control) was analyzed using the Student's t-test for independent samples, Mann-Whitney for numerical variables, and the χ2 test or Fisher's exact test for categorical variables. The comparison of treatment response (success and failure) between the groups was analyzed using the χ2 test.

## RESULTS

**Figure 4:**
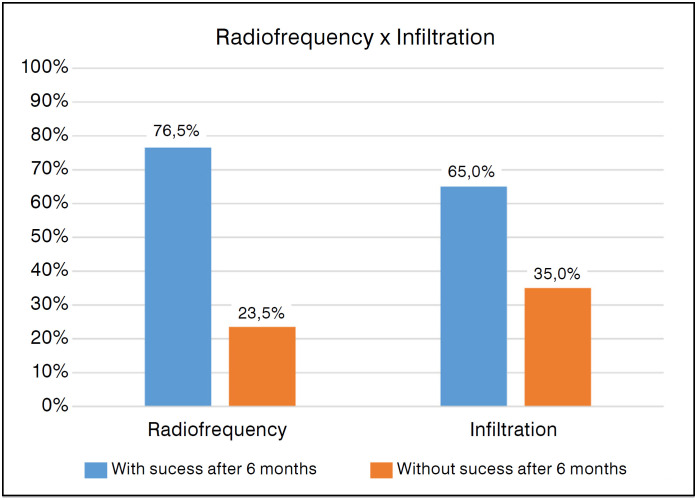
Comparative result of the VAS between the study and control groups after 6 months.

Source: Author's collection (2024).

The RF group exhibited a higher prevalence of individuals with a tendency towards valgus angle than the control group (17.6% vs 0%), p = 0.033. There was no statistical difference in the other variables between the groups. The RF group showed success on the VAS scale in the 1st month (74.5%) and in the 6th month (76.5%), significantly higher than the infiltration group (30.0% and 35.0%, p=0.0005 and p=0.0001) respectively. In the RF group, the average VAS varied from 8.8 to 3.2 (63% variation), while the IAC group varied from 8.9 to 5.8 (35%) at the 6th month. The RF group was superior to the control group with an odds ratio of 2.18 (Table 1 and Figure 4).

**Table 1:** Success of the treatment in the complete sample (VAS and WOMAC) and in the subgroups of WOMAC.

Variable		Total		Radiofrequency		Infiltration		*p value*	RR
		n	%	n	%	n	%		
**VAS Success 0-1 month**									
	yes	44	62.0	38	**74.5**	6	30.0	**0.0005**	**2.48**
	no	27	38.0	13	25.5	14	70.0		
**VAS Success 0-6 months**									
	yes	46	64.8	39	**76.5**	7	35.0	**< 0.0001**	**2.18**
	no	25	35.2	12	23.5	13	65.0		
**WOMAC Success 0-1 month**									
	yes	60	84.5	46	**90.2**	14	70.0	**0.044**	**1.29**
	no	11	15,5	5	9.8	6	30.0		
**WOMAC Success 0-6 months**									
	yes	60	84.5	46	**90.2**	14	70.0	**0.044**	**1.29**
	no	11	15.5	5	9.8	6	30.0		
**WOMAC Pain Success 0-6 months**									
	yes	61	85.9	47	**92.2**	14	70.0	**0.024**	**1.32**
	no	10	14.1	4	7.8	6	30.0		
**WOMAC Stiffness Success 0-6 months**									
	yes	13	18,3	13	**25.5**	0	0.0	**0.008**	*Undefined*
	no	58	81.7	38	74.5	20	100.0		
**WOMAC Function Success 0-6 months**									
	yes	58	81.7	44	86.3	14	70.0	0.10	1.23
	no	13	18.3	7	13.7	6	30.0		

Data are expressed as frequency (n) and percentage (%), Chi-square test or Fisher's exact test. RR: relative risk of radiofrequency for success.

Source: Own authorship (2024).

The RF group demonstrated success on the WOMAC at the 1st month (90.2%) and at the 6th month (90.2%) greater than the infiltration group (70.0%, 70.0%), with p=0.044, p=0.044.

**Figure 5:**
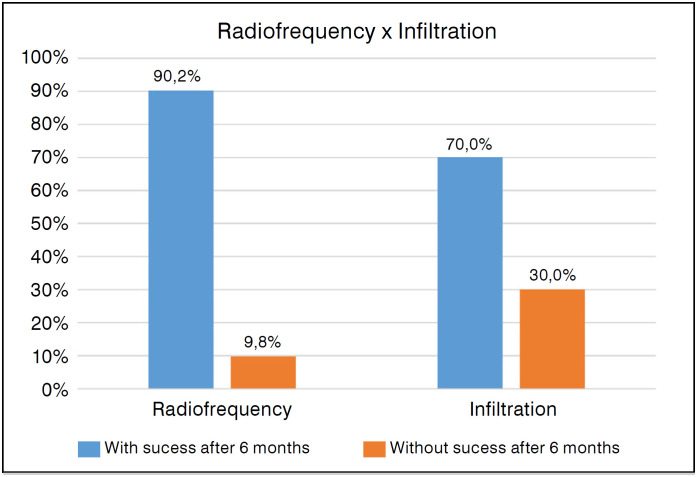
Comparative result of the WOMAC between the study and control groups after 6 months.

Source: Author's collection (2024).

**Figure 6:**
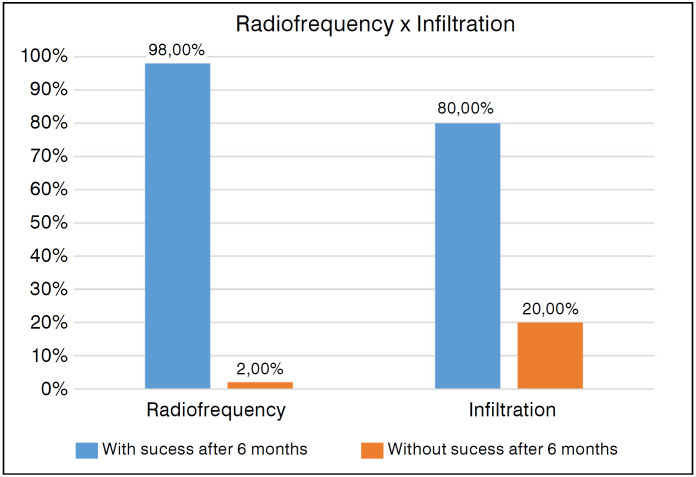
Comparative result of the KSS Knee between the study and control groups after 6 months.

Source: Author's collection (2024).

In the WOMAC subgroups, pain at the 6th month (92.2%), WOMAC stiffness at the 6th month (25%) showed superior performance compared to the control group (70.0% and 0%, p=0.024 and p=0.008) respectively (Figure 5). The WOMAC in the RF group decreased from 63.3 to 42.5 (37.5% percentage variation) in 6 months, while group 2 varied from 60.5 to 46.3 (20.96%). The RF group exhibited a success rate according to the KSS Knee at the 6th month (98.0%) and KSS Function at the 6th month (78.4%) significantly higher than the infiltration group (80.0% and 55.0%, p=0.020, and p=0.048) respectively (Table 2, Figure 6).

**Table 2:** Treatment success in the complete series relative to KSS Knee and KSS Function.

Variable		Total		Radiofrequency		Infiltration		*p value*	RR
		n	%	n	%	n	%		
**KSS Knee Success 0-1 month**									
	yes	66	93.0	50	**98.0**	16	**80.0**	**0.020**	**1.22**
	no	5	7.0	1	2.0	**4**	20.0		
**KSS Knee Success 0-6 months**									
	yes	66	93.0	50	**98.0**	16	**80.0**	**0.020**	**1.22**
	no	5	7.0	1	2.0	**4**	20.0		
**KSS Function Success 0-1 month**									
	yes	49	69.0	38	74.5	11	55.0	0.11	1.35
	no	22	31.0	13	25.5	9	45.0		
**KSS Function Success 0-6 months**									
	yes	51	71.8	40	**78.4**	11	**55.0**	**0.048**	**1.42**
	no	20	28.2	11	21.6	9	45.0		

Data are expressed as frequency (n) and percentage (%). Chi-square test or Fisher's exact test. RR: relative risk of radiofrequency for success.

Source: Author's arquives

In the RF group, a high BMI (>30) correlated with a favorable prognosis with improvement on the VAS scale and the WOMAC scale subgroup regarding pain response at the end of 6 months (X2 test p=0.03 and p=0.019, respectively).

Knee angulation in valgus demonstrated a negative response to the RF method when evaluated by the VAS scale, total WOMAC, and WOMAC in the pain subgroup (X2 test p=0.032, p=0.040 and p=0.028, respectively). The degree of osteoarthritis assessed by the Kelgren and Ahlback scales showed a relationship between increased radiographic severity and improvement in the stiffness component response of the WOMAC at the 6th month (X2 test p=0.019, p=0.037, respectively). The other parameters did not have prognostic relevance. The remaining parameters did not show prognostic relevance.

It was noted that in the RF group, non-obese individuals had a significantly higher prevalence of genu valgum cases (p<0.001) than obese individuals. As previously shown, this deformity would be linked to inferior functional outcomes. The good results of the obese group could be explained by the lower prevalence of genu valgum cases and not necessarily by the BMI itself.

No clinical complications were detected with the RF technique.

## DISCUSSION

The main finding of this study was that the RF group achieved better results in pain control and in the functional scales WOMAC and KSS than the group treated with corticosteroids at the sixth month of follow-up. It was evidenced that factors such as the severity of osteoarthritis and the angulation of the knee in valgus influenced the outcome of pain response and/or functionality.

In this study, the main indication for RF occurred in patients selected for TKA, who were fit and willing to undergo surgery, however, without the availability of the implant. Such a situation creates a bias that challenges the RF method, as the patient is fully aware that there is a gold standard, namely, the prosthesis.

No test anesthetic block was used to determine the indication for the RF procedure due to its low prognostic value^19,20^.

The authors used the image intensifier to perform the procedures. There was a disadvantage of exposure to radioactivity compared to the use of ultrasound. Kim et al^14^ did not demonstrate differences in outcomes between the techniques.

Juni et al^10^ evidenced improvement in pain up to the sixth week after corticosteroid infiltration for knee arthritis. After six months, there would be no influence on the maintenance of joint space or improvement in quality of life.

In 2016, Sari et al^1^ conducted the first randomized study on knee arthritis using conventional RF compared to the infusion group with corticosteroids. At the end of three months, the RF group showed improvement in pain (VAS) and a decrease in WOMAC.

Davis et al^2^ in 2018 compared cooled RF with intra-articular corticosteroid infiltration in knee arthritis. There were 76 treated with RF and 75 with corticosteroids. At the sixth month, a reduction of more than 50% in pain intensity occurred in 74.1% of the study group versus 16.2% of the control. In the current research, the results obtained were similar to those of Davis et al^2^ and Sari et al^1^. The functional gains obtained tended to be maintained from the first to the sixth month, similar to other authors^17,21^.

Recent meta-analyses^2,13^ identified sustained improvement in pain levels and the WOMAC scale of the RF groups compared to the control groups (hyaluronic acid, corticosteroid, placebo), not reporting serious adverse effects similar to the present study.

In this research, the authors utilized conventional cannulas. The cooled RF tip provides a volumetric lesion 5 to 20 times greater than the traditional tip^22,23^. Some authors^22,24^ argue that cooled RF would achieve better and more lasting results than the conventional method. However, a meta-analysis^25^ addressing cooled, pulsed, and conventional RF did not demonstrate a difference in pain improvement up to 12 months post-procedure. This observation reinforces the use of conventional RF, a technique more likely to be employed in public programs for palliative pain management.

The radiographic alignment of the limb had not yet been studied in the researched literature regarding its impact on the outcome of RF therapy. It was found that patients with genu valgum would exhibit a lower response regarding pain improvement (VAS) and function (WOMAC). Genu valgum generally presents as a more complex pathology than genu varum for surgical approaches. However, in the case of knee RF, an explanation for the poorer results could be the limited number of points addressed by the method on the lateral side, which would be the predominantly painful side. In this study, only one lateral point was treated. Chen et al^25^ demonstrated that the more points addressed in the knee, the better the response. The approach to the recurrent branch of the fibular nerve on the lateral tibia could be an option that improves the response to RF. The severity of genu varum deformity was not related to the clinical response to the RF method.

In the initial analysis of the results, obesity (BMI>30) appeared to be a positive prognostic factor for pain improvement (VAS scale). Such a finding would be corroborated by Chen et al^25^. On the other hand, Santana Pineda et al^26^ described elevated BMI as a factor for inferior response to RF. After comparing the obese and non-obese groups in RF, a lower prevalence of genu valgum cases was identified among the obese, which initially led to a mistaken conclusion regarding obesity and good prognosis. In fact, the smallest number of cases of valgus in the present cohort of obese individuals could be a more plausible explanation for this result. According to Kapural et al^6^, the BMI would not have a relationship with the outcome of RF therapy.

This study demonstrated that individuals treated with RF and with more advanced radiographic knee arthritis showed functional improvement measured by the WOMAC stiffness scale. Sari et al^1^ and Caragea et al^27^ had already reported similar observations. The Alhback classification, widely used by orthopedic surgeons, allows for staging with more elaborate severity types than the Kelgren classification^18^. No other article was found regarding RF using the Alhback classification. It should also be considered that the group studied here addresses severe cases, with a long evolution and with a well-established indication for TKA.

Recently, Dias et al^28^ published a randomized study conducted on patients with Kelgren IV knee osteoarthritis comparing the use of pulsed RF (17 patients) versus infiltration with phenol (18 individuals), demonstrating improvements in pain and function maintained until the third month of follow-up, without superiority of one methodology over the other.

The current research has as a critical factor the non-randomization of cases; however, the statistical analysis demonstrated that the RF and control groups were comparable and homogeneous, except for genu valgum, which was shown to be more prevalent in the RF group and related to inferior functional prognosis. Despite this, the RF group showed a response significantly superior to the control group regarding pain improvement and functional outcome. Another point would be the lack of evaluation of emotional state and opioid use. It is well established in the literature^25-27^ that depression and emotional disorders negatively influence outcomes. The reliable quantification of pain medication use in patients who frequently present with multiple pain sites is a difficult and unreliable task.^6^

Positive points include the fact that the study is prospective, involving patients with an indication for TKA who wish to undergo surgery. This fact generates a more demanding view regarding the RF method, which, combined with the use of measures such as the MCID, places the results under more rigorous scrutiny. Furthermore, the evaluation of parameters not previously studied, such as tibiofemoral alignment and the Ahlback classification, deserves emphasis.

The initial results have been encouraging. The research should continue aiming at the improvement of the technique, the use of new sites for RF, and the identification of the most suitable patient for the procedure through the determination of epidemiological, clinical, and radiographic factors with prognostic influence.

## CONCLUSION

The RF group exhibited greater improvements in pain and function compared to the control group. The severity of osteoarthritis and knee angulation influenced the functional and pain outcomes.

## Data Availability

The underlying contents of the research text are contained in the manuscript.
